# Human Endogenous Retrovirus K in Cancer: A Potential Biomarker and Immunotherapeutic Target

**DOI:** 10.3390/v12070726

**Published:** 2020-07-06

**Authors:** Gislaine Curty, Jez L. Marston, Miguel de Mulder Rougvie, Fabio E. Leal, Douglas F. Nixon, Marcelo A. Soares

**Affiliations:** 1 Programa de Oncovirologia, Instituto Nacional de Câncer, Rio de Janeiro RJ 20231-050, Brazil; fabio.leal@inca.gov.br; 2 Division of Infectious Diseases, Department of Medicine, Weill Cornell Medicine, New York, NY 10065, USA; jlm4001@med.cornell.edu (J.L.M.); mid2034@med.cornell.edu (M.d.M.R.); dnixon@med.cornell.edu (D.F.N.)

**Keywords:** HERV, HERV-K, HML-2 subtype, endogenous retrovirus, oncogenesis, cancer, neoantigen, tumor-specific antigens, immune response, immunotherapy

## Abstract

In diseases where epigenetic mechanisms are changed, such as cancer, many genes show altered gene expression and inhibited genes become activated. Human endogenous retrovirus type K (HERV-K) expression is usually inhibited in normal cells from healthy adults. In tumor cells, however, HERV-K mRNA expression has been frequently documented to increase. Importantly, HERV-K-derived proteins can act as tumor-specific antigens, a class of neoantigens, and induce immune responses in different types of cancer. In this review, we describe the function of the HERV-K HML-2 subtype in carcinogenesis as biomarkers, and their potential as targets for cancer immunotherapy.

## 1. Introduction

Endogenous retroviruses make up about 8% of the human genome [1]. They originated millions of years ago by retroviral infections in germline cells and currently remain in the human genome as “fossil” sequences [1,2,3].

There are several human endogenous retrovirus (HERV) families, as shown in Table 1, some of them composed of a full-length or almost complete retroviral genome, with *gag*, *pol* and *env* genes flanked by LTR regions. The *env* genes are commonly mutated and are therefore unable to produce infectious viral particles. However, HERV proteins synthesized by *env* transcripts play an important role in cellular regulation [2,3,4]. For instance, the *env* transcript from HERV-W is fundamental to the formation of the placenta during embryonic development [5].

The transcription of HERVs is mainly controlled by epigenetic mechanisms, such as the methylation of CpG regions [6,7,8,9]. HERV expression is inhibited in normal healthy adult cells [10,11]. However, in diseases where epigenetic mechanisms are altered, such as in cancer, HERV expression is upregulated and HERV proteins play an important role in carcinogenesis [3,7,12,13]. For example, *env* transcripts encoded by the HERV-K HML-2 subtype—hereafter abbreviated to HERV-K—the most studied HERV element, produce two oncogenic proteins (Rec and Np9) which are able to modulate cellular gene expression and induce cancer development [3,12]. Furthermore, its derived proteins could behave as tumor-associated neoantigens and induce immune responses in different types of cancer [3,4,12,14,15,16]. In this review, we describe the function of HERV-K in carcinogenesis, as well as its use as biomarkers and as targets for cancer immunotherapy.

## 2. HERVs: Classification and Genome

### 2.1. Nomenclature and Classification

Since HERVs were discovered, at least 31 distinct HERV groups have been described, with copy numbers ranging from one to many thousands in the human genome [17,18]. HERV group classification is based on the tRNA type used as primers during reverse transcription. However, the nomenclature system is not unified and there are multiple names for each unique HERV. HERV-K nomenclature is a good example of this inconsistency, as shown in Table 1 [17,19,20]. HERV-K has been described with multiple names, such as HLM-2, HML-2, HERV-K10, HTDV/HERV-K, HERV-K (HML-2), HERV-K, HERVK or ERKV. The letter “K” in the group name is based on the lysine-tRNA used during reverse transcription. In addition, the HERV-K group (HML1-HML10) can also be classified into type I and II proviruses, however phylogenetic type I and II classification from the HERV-K group is not the same for all HML subtypes. For instance, HERV-K HML-2, HML-6 and HLM-10 subtypes are classified into type I or II, based on differences from their genomes; however type I and II from each of them are unrelated [21,22,23,24]. Moreover, phylogenetic analysis using LTR sequences can be used to classify HERV-K, HML-2 subtype, into three subgroups, known as LTR5Hs, LTR5A and LTR5B [2,19,24,25]. The LTR5Hs is the most recently integrated subgroup. Interestingly, the HERV-K HML-2 types I and II are not equally distributed within LTR subgroups. While types I and II are found in equal frequency within the LTR5Hs subgroup, LTR5B and LTR5A subgroups show only the type II variant [24]. Due to this complex classification system, some authors have proposed a unified system of endogenous retrovirus nomenclature [20,26,27].

HERVs are phylogenetically similar to exogenous retroviruses due to their origin and can therefore be categorized into three retrovirus classes [28,29,30]. HERVs of Class I are similar to exogenous Gammaretroviruses; Class II to Betaretroviruses and Class III are distantly related to Lentiviruses and Spumaviruses, as shown in Table 1. Unlike exogenous retroviruses, HERV genomes show many mutations and deletions that prevent the production of infectious viral particles.

### 2.2. Genome Structure

HERV elements originated in the human genome through many insertion events of exogenous retroviruses in germline cells throughout evolution [3,31]. Over evolutionary time, HERV genomes suffered many mutations and this modification rate can be used to define the approximate time in the past of a particular HERV introduction into the human genome [2]. HERVs with recent introductions show full-length or almost complete sequence genomes, which are composed of *gag*, *pol* and *env* genes flanked by LTR regions [2].

The majority of HERV-related sequences in the genome are solitary LTR (solo LTR) sequences [32]. They were generated in the genome by homologous recombination between two LTRs flanking *gag*, *pol* and *env* genes, resulting in the deletion of these regions [33,34]. These HERV sequences do not produce viral proteins but are able to regulate cellular gene expression through their promoter regions [35]. In contrast, in full-length or almost complete genome HERV sequences, such as in HERV-K, the *gag, pol* and *env* genes produce their respective viral proteins, and non-infectious viral-like particles in diseases such as cancer [36,37,38,39]. The main *env* gene product is the Env protein, displaying a surface (SU) and a transmembrane (TM) domain. However, the HERV-K subtype encodes two alternative proteins, called Rec and Np9 by alternative splicing events.

**Figure 1:**
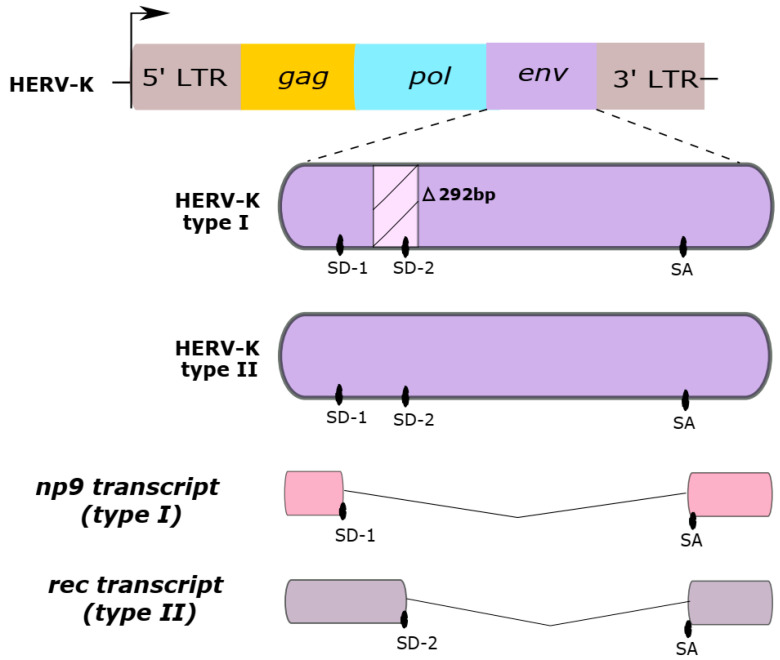
Two HERV-K HML-2 genome types. Type I sequences have a deletion in the *env* gene of a 292-base pair region (∆292pb). This region has a splice donor (SD) site 2, which is absent in the HERV-K type I. Type II sequences contain two splice donor sites (SD-1 and SD-2). Differences in the presence of the SD sites are responsible for generating the distinct np9 or rec transcripts from Type I and II sequences, respectively. SA, splice acceptor site.

HERV-K HML-2 subtype sequences are classified as type I or type II, according to either the absence or presence of a 292-base pair portion in the *env* coding region, as shown in Figure 1. While type II sequences are able to produce the viral accessory Rec protein, type I sequences cannot produce the Rec protein but are instead able to synthesize an alternative protein, called Np9. Both Rec and Np9 proteins have been postulated to be involved in carcinogenesis [3,19,36,40,41,42].

In healthy adult cells, HERV gene expression is inhibited by epigenetic regulation. In some diseases, such as cancer, the epigenetic mechanisms become dysregulated and many previously repressed genes become expressed, including HERV-K genes [43,44]. DNA methylation, the addition of methyl groups onto the cytidines of CpG regions in the DNA, and histone modification, such as the removal of acetyl groups of histones, cause chromatin condensation and make promoter regions inaccessible to transcription factors and to transcription machinery. In addition, the methylation of histones can also control HERV expression. SETDB1 (SET domain bifurcated histone lysine methyltransferase 1) is a histone methyltransferase, which methylates Lys-9 of histone H3, leading to transcriptional repression. SETDB1 is upregulated in various tumor cells and plays an important role on the silencing of endogenous retroelements [45,46]. These mechanisms contribute to the regulation of HERV expression [11,46,47,48,49]. HERV DNA methylation occurs in CpG regions of the LTR promoters and inhibits or downregulates HERV gene expression in normal cells [11,50,51,52]. The hypomethylation of HERV-K has been associated with poor ovarian cancer prognosis [53]. Interestingly, HERV-K also shows differential gene expression between individuals due to LTR polymorphisms at transcription factor binding sites [54].

The first investigations into the association of HERV expression with carcinogenesis were reported in research from the early 1970s exploring reverse transcriptase (RT) protein activity and viral particles in cancer cells [55,56]. Currently, HERV-K transcription is related to many kinds of cancer, such as breast cancer, melanoma and prostate cancer [57,58,59]. However, its gene expression in breast cancer and melanoma is the most studied as biomarkers and immunologic therapeutic targets [59].

In breast cancer, HERV-K RT expression is found in about 28% of samples and in 18% of adjacent normal breast tissues. There is also a significant correlation between HERV-K RT expression and poor prognosis for disease-free patients that go on to develop disease, suggesting HERV-K could be an early prognostic biomarker for breast cancer [60]. Furthermore, HERV-K *env*, *gag* and *np9* mRNA expression levels are also elevated in breast cancer cells and their use as biomarkers for early breast cancer diagnosis has been proposed [61]. The HERV-K *env* gene is expressed in 70% of breast cancers and its expression is associated with breast cancer progression [62]. HERV-K *env* gene expression was associated with tumor size, tumor stage, and lymph node metastasis. Furthermore, breast cancer patients with high HERV-K *env* expression show decreased overall survival compared to patients who had tumors with moderate or low HERV-K expression [62]. HERV-K *env* expression was not detected in normal breast tissues, suggesting that its expression is absent in the normal tissues. In addition, HERV-K was significantly overexpressed in basal breast cancer subtypes—the breast cancer subtype with the worst prognosis [63]. Finally, HERV-K *gag* mRNA overexpression has been reported in breast cancer patients who developed metastatic tumors when compared with those with tumors that did not metastasize [64]. Similarly, melanoma also shows high HERV-K gene expression, along with the production of retrovirus-like particles in tumor cells [65,66].

**Figure 2:**
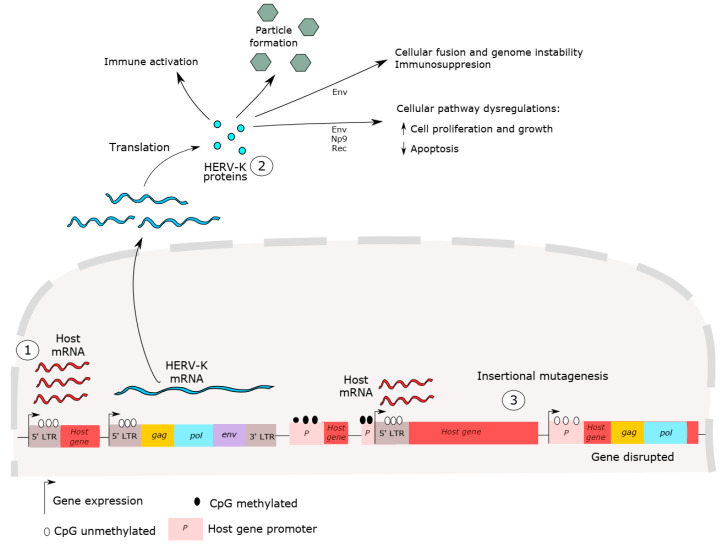
HERV-K carcinogenesis mechanisms. (1) Dysregulation of host gene expression by LTR promoter sequences. HERV LTR may influence neighboring host gene expressions, such as those of oncogenes, proto-oncogenes and growth factors. (2) HERV proteins can induce immune activation and suppression, lead to cell fusion, genome instability and cellular dysregulation. (3) HERV-K insertion mutagenesis, induced by recent retrotransposition events, is also able to cause host gene alterations, such as disruption in host genes, inducing host gene expression and causing genome instability. No HERV-K copies are competent for genomic reinsertion, but HERV-K insertional polymorphisms exist in the human population, suggesting these elements might provide a platform for genomic rearrangement. In short, all these events are able to disrupt cellular processes and lead to cancer initiation and progression.

Many studies have discussed the mechanisms of carcinogenesis mediated by HERV-K expression, as shown in Figure 2 [2,12,67]. However, whether HERV has a role in cancer initiation or cancer progression is still controversial. HERV-K insertional polymorphisms, responsible for HERV haplotype diversity in the human population, can influence disease susceptibility, including cancer [40,68,69,70,71]. HERV-K LTR sequences are able to up- and downregulate host genes [72]. Host gene expression dysregulation, such as that of oncogenes, proto-oncogenes and growth factors has been reported in cancer and has been associated with HERV LTR promoter activity [35]. These sequences may influence the expression of neighboring host genes and act as alternative promoters or enhancers of host genes [35,73,74]. For example, hypomethylation in LTR promoters is able to induce carcinogenesis in B cell-derived Hodgkin’s lymphoma by deregulating the expression of the colony-stimulating factor 1 receptor (CSF1R), a proto-oncogene [75]. In addition, despite the high HERV-K expression in cancer, de novo insertion by re-infection events has not been detected, which is not surprising since no HERV-K copies are known to be retrotransposition competent.

HERV-K proteins have also been shown to interact with host proteins and lead to cancer progression [76]. Intriguingly, the HERV-K Env protein exhibits many functions, including cancer cell fusion and host immunosuppression, as shown in Figure 2. Cancer cells are able to fuse with other cells, leading to chromosomal instability. This process may be associated with cancer progression and metastasis and chemoresistance [77,78]. Additionally, the immunosuppressive activity of the Env protein may lead to a tumor’s ability to evade immune responses, through the inhibition of the CD8-T cell cytotoxic activity against cancer cells and the prevention of apoptotic responses [79,80]. This property is co-opted in the fetal–maternal tolerance promoted by the expression of Syncitin 2, a HERV-FRD-derived Env protein. This property of Syncitin 2 led to the identification of an immunosuppressive domain (ISD) in the transmembrane region of the Env protein. Further studies exposing human PBMCs to ISD-derived proteins from HERV-K and other retroviruses, such as HIV, showed increases in the expression of numerous immunomodulatory factors, such as IL-10, IL-6 and IL-8, with decreases in the expression of the immune stimulatory factors IL-2 and CXCL9. However, the overall effect of this modulation remains to be clarified [81]. Furthermore, Env proteins produced by HERV-K mimic the oxygen response element binding protein (OREBP), affecting glutathione peroxidase expression and resulting in increased levels of free radicals in melanoma cells [82]. In vivo, HERV-K *env* RNA knockdown led to reduced metastasis [83]. Finally, the HERV-K Env protein is able to affect cellular networks and tumor-associated gene expression that play key roles in carcinogenesis (EGFR, c-Myc, TGFB1, NF-κB, p53, p-ERK, p-RSK, p-AKT and Ras) [76,83,84]. In particular, the HERV-K *env* gene can also produce Np9 or Rec proteins through alternative splicing from the *env* transcript.

### 2.3. HERV-K Oncoproteins

#### 2.3.1. Rec

Rec is a 14.5 kDa protein with functional homology to HIV-1 Rev and HTLV Rex proteins, which are responsible for translocating both partially spliced and unspliced retroviral transcripts from the cellular nucleus to the cytoplasm [23,85]. HERV-K RNA transport is mediated by Rec protein binding to the Rec-responsive element (RcRE) that is located within the LTR sequence on the 3′ end of unspliced viral RNAs [19,86].

The description of Rec expression in cancer was first reported in human germ cell tumors [87]. Following this, it was shown that nude mice that received a cell line expressing Rec eventually developed cancer, but not mice treated with cells expressing the full-length *env* or *gag* genes [88]. Supporting these findings, a study found that transgenic mice expressing Rec were able to develop in situ testicular carcinomas and predecessor lesions [89]. Moreover, in breast cancer, anti-Rec antibodies were detected in early-stage patients, suggesting a predictive biomarker for breast cancer progression [64].

The Rec protein has been shown to interact with zinc-finger proteins, such as the tumor suppressor, promyelocytic leukemia zinc-finger protein (PLZF) [88], associated with leukemia development. The Rec binding for PLZF leads to the higher expression of the *c-myc* proto-oncogene, consequently stimulating cell growth and proliferation [90]. Rec is also able to interact with the androgen receptor (AR), PLZF-related testicular zinc-finger protein co-repressor (TZFP) and with the human small glutamine-rich tetratricopeptide repeat-containing protein co-chaperone (hSGT), forming the complex Rec/AR/TZFP/hSGT [91,92]. This Rec-containing complex might lead to carcinogenesis by inducing cellular proliferation and reducing apoptosis [91,93].

#### 2.3.2. Np9

Np9 is a 9kDa protein that shares a region of 14 amino acids (MNPSEMQRKGPPRR) with the Rec protein in its N-terminal portion [90,94]. Np9 is also able to bind to PLZF in the nucleus, interfering with c-Myc repression in a similar fashion to Rec [90]. However, Np9 has also been shown to interact with E3 ubiquitin ligases, such as the ligand of numb protein X (LNX) and the murine double minute 2 (MDM2) protein, which are involved in the proteasome-dependent degradation pathways [95,96,97]. MDM2 has an essential function in the negative regulation of P53 through its degradation by ubiquitination. Therefore, dysregulation in this pathway leads to cell cycle dysfunction and is able to promote cellular proliferation and cancer initiation [97]. Finally, the expression of Np9 in leukemia cells is able to activate leukemia-associated signaling pathways and induce alteration in pERK, c-Myc and β-catenin expression, each of which has been shown to be altered in cancer cells [98].

## 3. HERV-K in Cancer Immunotherapy

HERV proteins are characterized within the group of alternative tumor-specific antigens, a neoantigen class, due to their expression in many kinds of cancer [4,99]. Tumor-specific antigens are defined as peptide antigens expressed in cancer cells and with minimal to no expression in normal healthy adult cells, as is the case of the expression of HERV-K antigens [99]. They are able to impact both innate and adaptive immune responses through distinct mechanisms. HERVs can induce the innate immune response by RIG-I-like and Toll-like receptor pathways through HERV nucleic acids [4,100,101,102]. The RIG-I-like and Toll-like are pattern recognition receptors able to recognize conserved pathogen-associated molecular patterns, such as ssRNA and dsRNA from viruses. RIG-I receptors mediate antiviral signaling via CARD–CARD interactions with the mitochondrial outer-membrane-localized adaptor molecule through mitochondrial antiviral signaling (MAVS). Such signal transduction leads to type I IFN induction and pro-inflammatory cytokine production, via the activation of IFN regulatory factors 3 (IRF3), and induce nuclear factor κB (NF-kB) expression [4]. Both pattern recognition receptors can induce inflammation that causes immune activation and the expression of class I MHC on tumor cells. In short, the innate immune response activation leads to B- and T-cell stimulation, inducing antibodies and cytotoxic T cell responses [64,103,104,105]. Thus, proteins encoded by the HERV-K *env* gene are immunogenic and humoral and cellular responses against these HERV-K have been described [67].

Antibodies to HERV-K were shown to inhibit cancer growth in vitro and in animal models [106]. In conjunction with a dendritic vaccine, HERV-K Env antigens demonstrated in vitro activity in ovarian and breast cancer [16,107]. Additionally, new modalities, such as CAR-T cells, have shown novel potential for HERV-related cancer immunotherapy. By using the Sleeping Beauty system, HERV-K Env-specific chimeric antigen mouse monoclonal antibodies were inserted into CAR-T cells and showed anti-tumor activity in vitro [108]. Finally, a recombinant vaccine using modified Ankara virus, expressing HERV-K Env glycoprotein (MVA HERV-K Env), demonstrated activity in vitro and in animal models [109]. However, concerns remain about the possible safety issues of vaccinating patients against endogenous HERV antigens, given the possible roles of these gene products in normal physiological function [67].

Of note, the homology between the HERV-K-MEL protein, Bacillus Calmette–Guerin (BCG) and yellow fever virus vaccine has been described [110]. Interestingly, a case-control study showed that the BCG vaccine was associated with lower melanoma risk in patients compared to the unvaccinated population [82]. Similarly, immunoreactivity to melanoma has been described in vitro with sera from rhesus macaques, vaccinated with the yellow fever virus vaccine, and this vaccine has been proposed as a prophylactic vaccine against melanoma. Given HERV over-expression, noted following BCG, yellow fever virus vaccine and the post-febrile process in melanoma patients, there is strong evidence to suggest that HERV-K gene expression may play a role in anti-melanoma immunoreactivity [111]. Additionally, the dependency of Env expression from a single provirus in a subset of individuals and a pattern of tissue-specific expression among proviruses in Mantle Cell lymphoma cell lines implies that HERV-K-targeted immunotherapy could be a precision medicine technique to specifically target the cell-specific aberrant transcription of this tumor-associated antigen in blood cancers. This could lead to a more targeted proteome-based screening protocol for HERV-K polymorphisms in blood cancers [15]. In short, all these studies show HERV-K expression as a target for cancer immunotherapy.

Several studies have reported humoral and cell-mediated immunity against HERV-K in cancer, as shown in Table 2. Breast cancer, melanoma and prostate cancer are the most studied types of cancer with HERV-K expression as new target in cancer immunotherapy, yet other types have also been studied in that regard.

### 3.1. Melanoma

Cytotoxic CD8 T-cell responses against HERV-K and their ability to lyse melanoma cells in vitro were first reported in 2002 [112]. Melanoma patients from stages I to IV showed significant differences in the seroprevalence of anti-HERV-K antibodies when compared to healthy subjects [114]. The serological HERV-K reactivity was inversely correlated with both disease specific (stage I–IV) and overall survival (stage I–III), providing new prognostic information on the disease [115].

In addition, chimeric antigen receptor (CAR) T-cells have been developed against HERV-K Env protein (HERV-K Env-specific CAR+ T-cells), which were found to be overexpressed in melanoma samples. HERV-K Env-specific CAR+ T-cells were able to lyse tumor cells expressing HERV-K Env on their surfaces in vitro. Furthermore, these CAR+ T-cells decreased tumor burden and the number of metastatic lesions to the liver in a mouse xenograft model of metastatic melanoma [116].

### 3.2. Breast Cancer

Anti-HERV-K antibodies have also been detected in breast cancer patients [16,64], and peripheral blood mononuclear cells (PBMC) from breast cancer patients stimulated in vitro with HERV-K are able to induce T-cell responses, such as T-cell proliferation, IFN-γ production and proinflammatory cytokine secretion [16]. Cytotoxic T-cells respond to breast cancer cells that express HERV-K, suggesting that HERV expression can be used as tumor-associated antigens for activating both T-cell and B-cell responses [16].

Anti-HERV-K antibodies and HERV-K *gag* mRNA detection showed diagnostic value for early breast cancer detection in women and can be used as sensitive and specific biomarkers for screening tests [64]. The HERV-K antibody’s diagnostic performance was comparable to mammography screening and can be performed as an additional option for early detection in women with increased breast cancer risk [64]. Furthermore, higher levels of HERV-K *gag* mRNA were detected in serum from breast cancer patients who developed metastasis in comparison with patients that did not [64].

Monoclonal anti-HERV-K Env antibodies showed antitumor effects as therapeutics against breast cancer in vitro and in vivo [105]. They blocked the growth and proliferation of tumor cells through the activation of apoptotic signaling pathways and, consequently cellular death in vitro. Likewise, mice receiving xenografts treated with the antibodies showed a reduction in breast tumor growth compared to mice with no antibody treatment [105].

HERV-K Env-specific CAR+ T-cells generated through monoclonal anti-HERV-K Env antibodies inhibited tumor growth and showed cytotoxic activity against breast cancer cell lines in vitro [108]. A significant reduction in tumor growth and tumor weight was also observed in mice xenograft models for breast cancer. In addition, HERV-K Env-specific CAR+ T-cells also prevented breast cancer metastasis in those mice.

### 3.3. Prostate Cancer

Both anti-HERV antibodies and HERV mRNA have been reported as biomarkers for prostate cancer and antibody production has been discussed as potential cancer immunotherapy [118,119,123,124,125,126]. HERV-K *gag* mRNA expression in prostate cells is regulated by both HERV promoter demethylation and androgen stimulation [117]. Results have suggested that the combination of HERV-K *gag* expression with prostate-specific antigen (PSA) testing using blood samples may be efficient to detect early prostate cancer, specifically in older men and smokers who at higher risk of developing more aggressive prostate cancer [118].

A panel for the detection of autoantibodies, including those against HERV and three host proteins, has been tested for analyzing the potential of using these autoantibodies in the diagnosis of prostate cancer [119]. The results showed that the detection of the anti HERV-K Gag antibody along with other host antibodies was successful in differentiating cancer patients from healthy subjects [119]. Furthermore, the anti-HERV-K Gag antibody is more frequent in serum from patients with advanced prostate cancer (stage III–IV) when compared to patients with early prostate cancer (stages I–II) [117]. The presence of the anti-HERV Gag antibody in patients’ sera has also been correlated with worse disease survival [117].

## 4. Other Cancers

Several studies have reported high HERV-K expression, adaptive immune responses and HERV-K antibodies in germline cell cancers [53,89,107,120,127,128,129,130,131]. These tumors occur in the testes and ovaries. HERV-K *env* and *RT* expression was higher in ovarian cancer in comparison to normal adjacent tissues and blood from ovarian cancer patients showed HERV-K antibody reactivity [107]. Autologous in vitro stimulation of T-lymphocytes from ovarian cancer patients with HERV-K Env protein exhibited cytotoxic activity against ovarian cancer cells [107]. In addition, we previously showed the HERV-K Gag protein and T-cell reactivity to HERV-K in seminoma patients [127].

HERV-K *env* is expressed in about 20% to 80% of pancreatic cancer tissues but not in normal counterparts [83,121]. High levels of HERV-K antibodies and HERV-K viral RNA have been reported in plasma from pancreatic cancer patients, suggesting HERV-K expression as a biomarker and a tumor-associated antigen that may be used for diagnosis and cancer immunotherapy [83,121].

HERV-K expression has also been associated with hepatocellular carcinoma (HCC) progression and poor outcome. HERV-K expression is upregulated in HCC, which was significantly associated with cancer staging, cirrhosis and tumor differentiation [122]. Furthermore, HCC patients with high HERV-K expression levels showed a poorer overall survival compared to patients with lower expression. In addition, HERV-K expression in HCC showed a diagnostic accuracy value, with 74.7% sensitivity and 67.8% specificity, which may be used as HCC diagnostics and as prognostic biomarkers for the disease [122].

## 5. Conclusions

HERVs are retroviral fossil sequences in the human genome that originated millions of years ago through retrovirus infections in germline cells and they now compose about 8% of the human genome. There are several HERV families, some of them are composed of full-length or almost complete genome retroviruses, showing *gag*, *pol* and *env* genes flanked by LTR regions. The *env* genes are mutated and, therefore, unable to produce infectious viral particles. However, HERV proteins synthesized by *env* transcripts play an important role in cellular regulation attributed to many kinds of HERVs.

The HERV-K family, the most studied, is expressed in many types of cancer. The *env* gene can give rise to two oncoproteins derived by alternative *env* mRNA splicing, called Np9 and Rec. Both oncoproteins are able to induce carcinogenesis by the dysregulation of essential cellular pathways, leading to the inhibition of apoptosis and to cellular growth and proliferation. Additionally, HERV-K proteins are classified within the neoantigen class of alternative tumor-specific antigens. They are able to impact both innate and adaptive immune responses, inducing B- and T-cell stimulation and activation. This can then lead to specific antibody and cytotoxic T-cell immune responses in many kinds of cancer, including breast cancer, prostate cancer, melanoma and renal cell carcinoma, and could be used as an immunotherapeutic target in these cancers.

## Figures and Tables

**Table 1:** Classifications of human endogenous retroviruses (HERVs) and phylogenetic relationship to exogenous retrovirus families.

Class	Family	Genus
Class I	HERV-H, HERV-F, HERV-W, HERV-R, HERV-P, HERV-E, HERV-I, HERV-T, ERV-FTD, ERV-FRD	Gammaretrovirus
Class II	HERV-K (HML 1–10)	Betaretrovirus
Class III	HERV-L	Distantly related to Lentivirus and Spumavirus

**Table 2:** Studies that have shown HERV-K expression as a biomarker for cancer screening and as an immunotherapeutic target.

Cancer	Study (Year)	Approach	Main Findings	Reference
Breast cancer	Golan, M. (2008)	The HERV-K RT expression was examined in 110 paraffin sections from breast carcinoma patients.	HERV-K RT expression correlated with poor prognosis in disease-free patients that go on to develop disease, suggesting HERV-K could be an early prognostic biomarker for breast cancer	[60]
	Wang-Johanning, F. (2012)	Human breast tissues and peripheral blood mononuclear cells from breast cancer patients and health women were used to analyze anti-HERV Env antibody and T-cell immune responses.	Breast cancer patients show HERV-specific antibody and T-cell immune responses, as well as proinflammatory cytokine production. The HERV-K-specific CD8 T-cell immune response was able to lyse breast cancer cells expressing HERV-K Env.	[16]
	Wang-Johanning, F. (2012)	The antitumor effect from anti-HERV-K Env monoclonal antibody was analyzed in vitro by quantifying cellular growth and apoptosis in breast cancer cells. In vivo, the tumor growth was analyzed using a mouse xenograft breast cancer model.	Anti HERV-K Env antibody shows antitumor effect. The antibody was able to inhibit cellular growth and induce apoptosis from breast cancer cells in vitro and in vivo.	[105]
	Wang-Johanning, F. (2013)	HERV-K mRNA and anti-HERV-K Env antibody were analyzed in serum samples collected from healthy women and breast cancer women patients. ELISA assay and real-time PCR were used to detect the antibody titer and the levels of HERV-K mRNA, respectively.	Anti-HERV-K Env antibody shows a diagnostic value compared to mammograms. Besides, HERV-K *gag* mRNA and Gag antibody showed sensitivity and specificity to be used as screening test to early-stage breast cancer diagnosis.	[64]
	Zhou, F. (2015)	The chimeric antigen receptor (CAR) specific for HERV-K Env was generated using anti-HERV-K Env antibody. Its antitumor effect was evaluated in vitro and in vivo, using breast cancer cell lines and xenograft breast cancer models, respectively.	HERV-K CAR T-cells showed a tumor-specific cytotoxicity in breast cancer cell lines and in a xenograft mouse breast cancer model. HERV-K CAR T-cells were also able to prevent tumor metastasis.	[108]
	Johanning, G.L. (2017)	A total of 512 breast cancer samples (117 basal, 53 Her2-enriched, 212 Luminal A and 130 Luminal B) deposited in the Cancer Genome Atlas were used to analyze four HERV-K loci expressions (HERV-K108 (7p22.1), HERV-K109 (6q14.1), HERV-K113 (19p12b) and HERV-K115 (8p23.1)) in breast cancer patients.	Four HERV loci were upregulated in the basal subtype (poor prognosis breast cancer subtype). HERV-K Env expression was significantly overexpressed in basal tumors in comparison with other upregulated HERV-K genes.	[63]
Melanoma	Schiavetti, F. (2002)	Peripheral blood mononuclear cells from melanoma patients treated with MAGE peptides and that showed tumor regression were isolated for identification of the antigen recognized by their CD8 T-cells.	Melanoma patients vaccinated with MAGE peptides are able to develop cytotoxic CD8 T-cells against HERV-K and to lyse melanoma cells in vitro.	[112]
	Büscher, K. (2006)	Melanoma biopsies and serum samples from melanoma patients were collected to analyze the anti HERV-K antibody and *env*, *rec* and *np9* HERV-K expression.	Expression of both *env* and rec were detected in 39% of the melanoma samples and in 40% of the cell lines. The np9 was detected in 29% melanoma samples and in 21% of the cell lines. Anti-HERV-specific Env antibodies were also detected in melanoma patients, however anti HERV-K Np9 and Rec antibodies were not identified. Immunosuppressive Env protein activity and release of virus particles were reported in vitro.	[113]
	Humer, J. (2006)	Serum samples from healthy and melanoma patients from stage I to stage IV were used to analyze anti HERV-K antibodies in melanoma patients.	Serum samples from melanoma patients show statistically significant differences in seroprevalence of anti-HERV-K Env antibody when compared to healthy subjects.	[114]
	Hahn, S. (2008)	Serum samples from healthy and melanoma patients were used to analyze anti HERV-K Gag and Env antibodies	Melanoma patients showed anti-HERV-K Gag and Env antibodies levels in the sera. Besides, patients with Anti HERV antibody show a significantly decreased disease-specific overall survival (stage I–IV).	[115]
	Krishnamurthy, J. (2015)	Chimeric antigen receptor (CAR) specific to HERV-K Env (K-CAR) were analyzed to kill melanoma cells in vivo using mouse xenograft melanoma model.	HERV-K Env CAR T-cell showed significant antitumor effect in melanoma in vivo, reducing primary tumor and metastatic burden in the mouse xenograft model	[116]
Prostate cancer	Reis, B.S. (2013)	HERV-K *gag* expression was analyzed in vitro using tissues (normal and tumor) and cell line. Anti HERV-K Gag antibody was also analyzed using serum samples from prostate cancer patients and healthy subjects.	HERV-K *gag* expression was upregulated in prostate cancer tissues and its expression was regulated both by demethylation and by androgen stimulation. Anti-HERV-K Gag antibody was also most frequent in serum from patients with advanced prostate cancer (stage III-IV) when compared to early prostate cancer (stages I-II), and it was correlated with worse survival.	[117]
	Wallace, T. A. (2014)	A total of 429 blood samples from African–American and European–American healthy men (*n* = 135) and those with prostate cancer (*n* = 294) were used to evaluate HERV-K *gag* mRNA and Env protein expression by quantitative real-time PCR and immunohistochemistry, respectively.	HERV-K Env protein was upregulated in prostate patients; however African–American patients showed higher expression than European–American patients. High HERV-K *gag* expression showed 12.87 fold increased odds (95% confidence interval 6.3–26.25) of being diagnosed with prostate cancer in comparison to patients that showed lower expression. HERV-K *gag* expressions were also associated with older age and smoking status, factors associated with risk of more aggressive prostate cancer disease.	[118]
	Rastogi, A. (2016)	Serum samples from 93 prostate cancer patients and 37 healthy subjects were used to analyze the autoantibody detection panel containing ERG, AMACR, C-MYC and HERV-K Gag proteins.	ERG, AMACR, and HERV-K Gag autoantibody detection were able to differentiate prostate cancer patients from healthy subjects.	[119]
Germ cell tumors	Kleiman, A. (2004)	Serum samples from germ cell tumor patients and control donors were collected. The anti-HERV-K Gag and anti-HERV-K Env were detected and clinical analyses were performed	Anti-HERV-K antibodies were detected in 67% of patients. Serological response was associated with clinical manifestation and cancer therapy success. The antibodies may have an important positive prognostic value to chemotherapy.	[120]
Ovarian Cancer	Rycaj, K. (2014)	HERV-K expression was analyzed in blood, cancer and normal tissue samples from patients with ovarian cancer and benign diseases. The anti-HERV-K antibodies were investigated in blood samples. PBMC was isolated and in vitro HERV-K Env antigen stimulation was performed.	HERV-K expression was higher in ovarian cancer in comparison to normal and adjacent normal tissues. Moreover, RT protein activity and anti-HERV-K antibodies were detected in blood from ovarian cancer patients. The immune HERV-K-specific T-cells, generated through autologous dendritic cell stimulation by HERV-K Env antigens, showed T-cell proliferation and cytotoxic T-lymphocyte activity against ovarian cancer cells.	[107]
Pancreatic cancer	Li, M. (2017)	Pancreatic cancer cell lines, biopsy tissue and patient sera were used for HERV-K expression analyses, virus-like particle detection and knockdown of HERV-K *env* to analyze the role of HERV-K expression in pancreatic cancer. In addition, an in vivo model was used to analyze the effect of HERV-K knockdown.	HERV-K expression and RT activity were shown in pancreatic cells, cancer tissue and patient sera. Virus-like particles were observed in cell culture supernatants. Moreover, knockdown of HERV-K *env* expression downregulated the RAS-ERK-RSK signaling pathway, important for cancer progression. The findings suggested that HERV-K proteins can be used as biomarkers and as a target to cancer immunotherapy.	[83]
	Schmitz-Winnenthal, F.H. (2007)	A total of 130 pancreatic adenocarcinoma tumors and 23 control tissue samples were collected from patients with chronic pancreatitis and from cadaveric donors. Tumor-associated antigen expression of 10 genes, including HERV-K, was assessed by PCR.	HERV-K expression showed a relatively high prevalence, with positivity in 23% of cases, which may be a tumor-associated antigen candidate for specific cancer immunotherapy.	[121]
Hepatocellular Carcinoma (HCC)	Ma, W. (2016)	A total of 84 HCC and normal adjacent tissue samples were collected to detect HERV-K expression by quantitative real-time PCR and clinical correlation analysis was performed.	HEVR-K levels were significantly increased in HCC and were associated with cirrhosis, tumor differentiation and TNM staging. Higher HERV-K expression was reported with poorer cancer prognosis. In addition, HERV-K expression demonstrated diagnostic accuracy (74.7% sensitivity and 67.8% specificity), which may be used as a prognostic biomarker for HCC.	[122]
